# The role of different strain backgrounds in bacterial endotoxin-mediated sensitization to neonatal hypoxic–ischemic brain damage

**DOI:** 10.1016/j.neuroscience.2015.10.035

**Published:** 2015-12-17

**Authors:** E. Rocha-Ferreira, E. Phillips, E. Francesch-Domenech, L. Thei, D.M. Peebles, G. Raivich, M. Hristova

**Affiliations:** Perinatal Brain Protection and Repair Group, EGA Institute for Women’s Health, University College London, WC1E 6HX London, UK

**Keywords:** ABC, avidin–biotin conjugate, BSA, bovine serum albumin, DAB, diaminobenzidine, dUTP, deoxyuridine triphosphate, GFAP, glial fibrillary acidic protein, GFAP-IR, GFAP immunoreactivity, HI, hypoxia–ischemia, IL, interleukin, LPS, lipopolysaccharide, MCA, middle cerebral artery, PB, phosphate buffer, PFA, paraformaldehyde, SAL, saline, TNFα, tumor necrosis factor alpha, TUNEL, terminal deoxynucleotidyl transferase dUTP nick end labeling, 129SVJ, BALB/c, C57BL/6, CD1, FVB, hypoxia–ischemia

## Abstract

Genetic background is known to influence the outcome in mouse models of human disease, and previous experimental studies have shown strain variability in the neonatal mouse model of hypoxia–ischemia. To further map out this variability, we compared five commonly used mouse strains: C57BL/6, 129SVJ, BALB/c, CD1 and FVB in a pure hypoxic–ischemic setup and following pre-sensitization with lipopolysaccharide (LPS).

Postnatal day 7 pups were subjected to unilateral carotid artery occlusion followed by continuous 30 min 8% oxygen exposure at 36 °C. Twelve hours prior, a third of the pups received a single intraperitoneal LPS (0.6 μg/g) or a saline (vehicle) administration, respectively; a further third underwent hypoxia–ischemia alone without preceding injection. Both C57BL/6 and 129SVJ strains showed minimal response to 30 min hypoxia–ischemia alone, BALB/c demonstrated a moderate response, and both CD1 and FVB revealed the highest brain damage. LPS pre-sensitization led to substantial increase in overall brain infarction, microglial and astrocyte response and cell death in four of the five strains, with exception of BALB/c that only showed a significant effect with terminal deoxynucleotidyl transferase dUTP nick end labeling (TUNEL). Saline administration prior to hypoxia–ischemia resulted in an increase in inflammatory-associated markers, particularly in the astroglial activation of C57BL/6 mice, and in combined microglial activation and neuronal cell loss in FVB mice. Finally, two of the four strongly affected strains – C57BL/6 and CD1 – revealed pronounced contralateral astrogliosis with a neuroanatomical localization similar to that observed on the occluded hemisphere.

Overall, the current findings demonstrate strain differences in response to hypoxia–ischemia alone, to stress associated with vehicle injection, and to LPS-mediated pre-sensitization, which partially explains the high variability seen in the neonatal mouse models of hypoxia–ischemia. These results can be useful in future studies of fetal/neonatal response to inflammation and reduced oxygen–blood supply.

## Highlights

• Strain background plays a role in the response to hypoxia–ischemia.
• LPS sensitizes the immature brain to hypoxia–ischemia across several mouse strains.
• Vehicle injection may induce immune response and sensitization to hypoxia–ischemia.

## Graphical abstract

**Figure:**
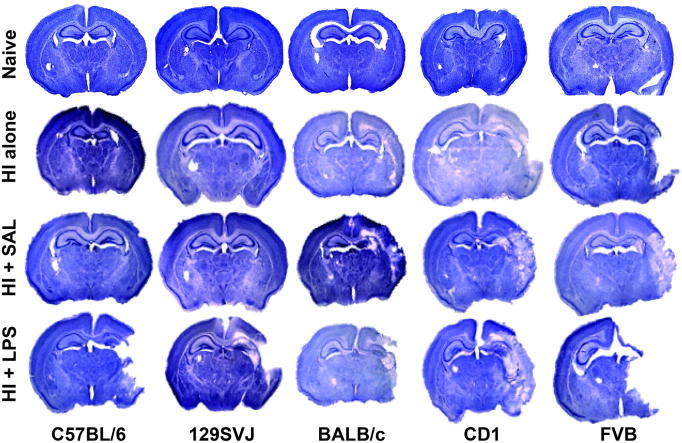

## Introduction

The postnatal day 7 mouse model of hypoxic–ischemic insult is widely used as a model of neuropathology that mimics, to a certain degree, the pathology of hypoxic–ischemic injury occurring in human late-preterm to term neonates (Clancy et al., 2007, Clancy et al., 2001, Sheldon et al., 1998). With the addition of transgenic mice, this model has become particularly relevant in establishing the genetic basis of injury evolution and assessing efficacy of experimental treatments. As clinical advances have shown perinatal brain damage to be multifactorial, the neonatal mouse model of hypoxia–ischemia encephalopathy has also been modified to include not just hypoxia–ischemia, but also an infection/inflammation-sensitizing element, known to exacerbate some human cases of perinatal brain damage (Eklind et al., 2001, Wu et al., 2003, Aly et al., 2006).

Previous research have shown that the strain background influences the degree of brain damage in neonatal mouse models of hypoxic (Li et al., 2013, Li et al., 2009, Li et al., 2008), ischemic (Comi et al., 2009) and hypoxic–ischemic (Sheldon et al., 1998) insults. Additionally, studies looking at inflammatory response in different mouse strains demonstrated that strain background plays a role in cytokine synthesis (Lambertsen et al., 2002, Hoover-Plow et al., 2008, Gibb et al., 2011, Browne et al., 2012) as well as inflammatory cell recruitment (White et al., 2002, Hoover-Plow et al., 2008). However, to our knowledge, no study to date has investigated whether this strain-mediated variation in response to injury is also seen in the synergistic model of inflammation and neonatal hypoxia–ischemia.

Addressing the variability in strain-mediated response to brain injury is paramount to improve understanding of the pathways and mechanisms involved in perinatal brain damage. In the current study, we have therefore used five different mouse strains – C57BL/6, 129SVJ, BALB/c, CD1 and FVB, commonly used as wild type and/or as source of transgenic animals – to investigate the effects of strain background in the mediating vulnerability or resistance to both hypoxia–ischemia alone and in endotoxin-mediated hypoxia–ischemia injury. For the latter, we used the well-established lipopolysaccharide (LPS) pre-sensitization to subsequent hypoxia–ischemia injury model (Hagberg et al., 2002, Wang et al., 2009, Kendall et al., 2011, Lange et al., 2014).

## Experimental procedures

### Animals

All animal experiments were carried out in accordance with the UK Animals (Scientific Procedures) Act 1986 and approved by the Home Office. The several lines of wild type mice used in this study: C57BL/6, 129SVJ, BALB/c, CD1 and FVB, were sourced from Charles River, UK. The ARRIVE guidelines were followed. All five different background strains were bred simultaneously, and experimental procedures carried out around the same period of time.

### Hypoxia–ischemia surgery and endotoxin

Postnatal day 7 mice were anesthetized with isoflurane (5% induction, 1.5% maintenance). The left common carotid artery was permanently ligated with 8/0 polypropylene suture and the incision closed using tissue glue. Pups were allowed to recover at 36 °C and returned to respective dams to nurse for 1.5 h before being placed in a sealed hypoxic chamber and exposed to a constant flow of humidified 8% oxygen/92% nitrogen at 36 °C for 30 min. After 10 min recovery at 36 °C, the mice were returned to their dams. 12 h prior, pups were randomly allocated to receive a single intraperitoneal injection of 0.6 μg/g LPS (*Escherichia coli*, serotype 055:B5; Fluka, UK), 0.9% sterile normal saline, or no treatment. All three experimental groups – HI alone, HI + SAL, HI + LPS – were performed simultaneously, ensuring all three groups contained littermates.

### Histochemistry and immunohistochemistry

Forty-eight hours after hypoxia–ischemia, animals were terminally anesthetized using pentobarbital sodium. Additionally, naïve animals (no surgery/hypoxia/injection, *n* = 3 per strain) were also perfused at P9 for brain histology. Paw withdrawal reflex was used to determine extent of anesthesia, before transcardial perfusion with 4% paraformaldehyde (PFA) in 0.1 M phosphate buffer (PB). Brains were extracted and post-fixed in 4% PFA/PB on a rotator for 1 h, followed by overnight rotation immersion in 30% sucrose for cryoprotection. The forebrains were frozen on dry ice and sectioned using a cryostat. Fifty sequential coronal sections of 40-μm thickness, starting from the fusion point of the corpus callosum, were collected onto 0.5% gelatinised glass slides and stored at −80 °C.

Tissue samples were stained as previously described (Hristova et al., 2010). In brief, brain sections were rehydrated in bidistilled water and spread flat onto the slide under a dissection microscope. After fan drying for 10 min, sections were fixed 5 min in 4% PFA and transferred into 0.1 M PB, treated with acetone (50%, 100%, 50%), washed twice in 0.1 M PB, and once in 0.1% bovine serum albumin (BSA)/0.1 M PB.

For immunohistochemistry, sections were blocked in 5% goat serum (Sigma, Gillingham, Dorset, UK) in PB for 30 min, and incubated with alphaM (αmβ2 integrin; 1:5000; Serotec, Kidlington, Oxford, UK) or glial fibrillary acidic protein (GFAP; 1:6000; DAKO, Ely, Cambridgeshire, UK) overnight at 4 °C. Both alphaM and GFAP immunostainings routinely included 2–3 negative control sections where respective primary antibody was absent. Sections were washed by serial immersions in PB/BSA, PB, PB and PB/BSA. The appropriate biotinylated secondary antibody (anti-rat or anti-rabbit) was pre-incubated with mouse serum (1:50; Serotec, Kidlington, Oxford, UK) for 30 min at 37 °C before being diluted to 1:100 in PB/BSA and sections incubated for 1 h at room temperature, washed twice in PB/BSA and PB, incubated for 1 h with avidin–biotinylated horse radish peroxidase Complex (ABC; 1:100; Vector, Peterborough, UK) solution, washed in four changes of 10 mM PB and visualized with diaminobenzidine (DAB; Sigma, Gillingham, Dorset, UK) in hydrogen peroxide (Sigma, Gillingham, Dorset, UK). Reaction was stopped in 10 mM PB and washed twice in bidistilled water. Slides were dehydrated by consecutive 2 min immersion in increasing concentrations of ethanol (70%, 90%, 100%), isopropanol, 3 times xylene, and covered using DEPEX.

For terminal deoxynucleotidyl transferase dUTP nick end labeling (TUNEL) assay, sections were incubated in 3% hydrogen peroxide in methanol (15 min), and washed in 0.1 M PB. Slides were then incubated with terminal deoxytransferase (TdT) and deoxyuridine triphosphate (dUTP) solution (0.1% TdT, 0.15% dUTP, 1% cacodylate buffer; Roche, Welwyn Garden City, Hertfordshire, UK) for 2 h at 37 °C. The reaction was stopped in TUNEL stop solution (300 mM NaCl, 300 mM Sodium Citrate) for 10 min. Slides were washed in 3 × 0.1 M PB, incubated in ABC solution (1 h at room temperature), washed 4 times in 10 mM PB and visualized using DAB enhanced with cobalt nickel in the presence of hydrogen peroxide. Reaction was stopped as described above.

Sections used for Nissl staining were fixed overnight in 4% PFA followed by 70% ethanol overnight. On day 3 sections were immersed in Cresyl Violet solution (BDH, East Grinstead, West Sussex, UK) for 3 min, followed by immersion in consecutive ethanol dehydration (70%, 90%, 96%, 96% with acetic acid, 100%), isopropanol and three washes in xylene before being covered in DEPEX.

### Histological assessment

Each histological assessment was performed on five sections 400 μm apart. Infarct size was measured using Optimas 6.5 image software, and the intact areas of the isocortex, pyriform cortex, hippocampus, striatum, thalamus and external capsule white matter were delineated bilaterally and measured as described previously (Kendall et al., 2012). Injury score (see Table 1) based on combined microglial activation (αM immunoreactivity) and neuronal cell loss (Nissl stain) was assessed in the same aforementioned brain regions and adapted from a scoring system previously established in our laboratory (Kendall et al., 2006). The presence of cell death involving DNA fragmentation was detected through quantification of the number of TUNEL positive nuclei in three separate fields (×20 magnification) per assessed brain region. Mean and standard deviation of optical luminosity values were measured in three fields (×20 magnification) of the different brain regions of GFAP-stained slides using Optimas 6.5 image software (Kendall et al., 2011). All assessments were performed by an observer blinded to the different treatments. Additionally, each assessment was performed for each of the strains simultaneously.

### Statistical analysis

Average ± standard error of the mean (SEM) was recorded for all data. Statistical analysis for infarct size, alphaM immunoreactivity and neuronal loss, TUNEL+ counts and GFAP luminosity between ipsilateral and contralateral brain hemispheres of all three different groups was initially tested for normal distribution. As the data did not follow the Gaussian distribution, the different assessments were performed using Kruskal–Wallis one-way analysis followed by Dunn multiple comparison test in PRISM GraphPad 6. Interstrain variations in response to HI alone, HI + SAL and HI + LPS were assessed for heterogeneity in meta-analysis using Chi-Squared test. *p* < 0.05 was considered to represent a significant difference between groups. In order to achieve the necessary statistical power, and based on previous studies performed in our group (Kendall et al., 2011, Lange et al., 2014), the size of the groups was adjusted to *n* = 10.

## Results

In the current study we used a combination of two different established models of neonatal mouse hypoxic–ischemic injury, known to generate ipsilateral brain damage in rats (Rice et al., 1981) and mice (Hagberg et al., 2004, Wang et al., 2009): (i) hypoxia–ischemia (HI) alone, where postnatal day 7 animals underwent unilateral left carotid artery occlusion followed by 30 min continuous 8% oxygen exposure at 36 °C, shown previously in our group to generate mild but definite ipsilateral damage in the C57BL/6 mouse strain (Kendall et al., 2011); and (ii) synergistic infection/inflammation and hypoxia–ischemia injury, using LPS pre-sensitization to subsequent hypoxia–ischemia, where LPS administration 12 h prior to hypoxia–ischemia insult is known to induce a robust pre-sensitization effect in the C57BL/6 mouse strain (Kendall et al., 2011, Lange et al., 2014). Saline (vehicle) pre-treatment was used as control for LPS administration, and untouched littermates prior to hypoxic–ischemic insult as control for any pre-hypoxia–ischemia intervention.

### Infarct volume

**Fig. 1:**
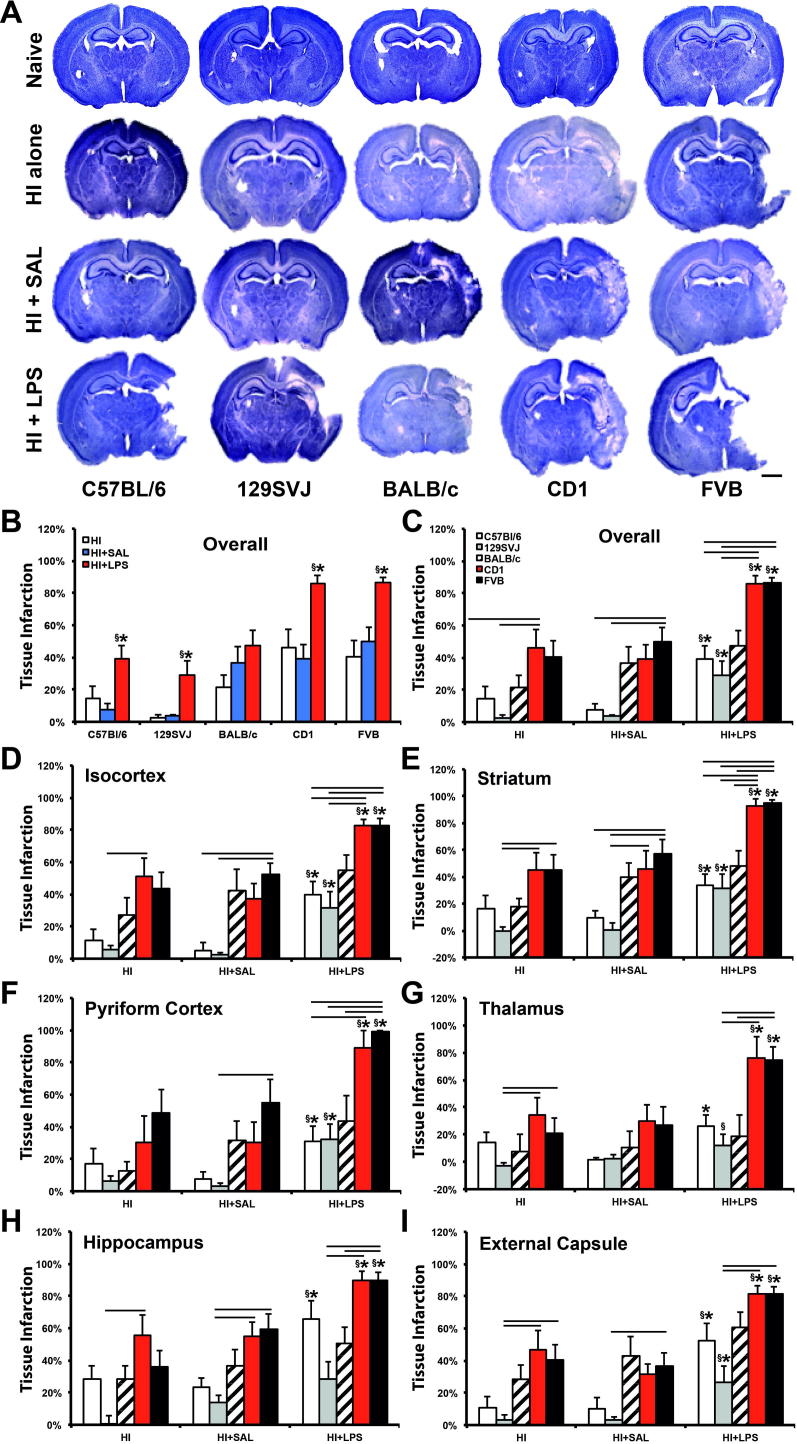
Pre-treatment with LPS 12 h prior to hypoxia–ischemia in postnatal day 7 mice normally results in augmented brain infarction across different mouse strains. (A) Nissl staining of mouse forebrains of C57BL/6, 129SVJ, BALB/c, CD1 and FVB strains 48 h following hypoxia–ischemia. Coronal hemisphere sections (40 μm) at the level of the cortex and beginning of the hippocampus; the upper row shows untreated naïve animals, second row mice exposed to hypoxic–ischemic insult alone (HI alone), third row those injected 12 h earlier with saline (HI + SAL), and lower row with pre-treatment of 0.6 μg LPS/g body weight (HI + LPS). (B, C) Infarct volume assessment of overall tissue loss per strain (B) and per treatment (C). (D–I) Individual forebrain regions in isocortex (D), pyriform cortex (F), hippocampus (H), striatum (E), thalamus (G) and external capsule (I) as percentage of that region on the contralateral hemisphere. Just minimal tissue loss was observed in 30 min hypoxia–ischemia alone and the HI + SAL groups in the mouse strains of C57BL/6 (*n* = 10 and 11 animals, respectively) and 129SVJ (10/10), but this increased, moderately, in the BALB/c (11/9), CD1 (10/12) and FVB (9/11). LPS pre-treatment strongly sensitized to hypoxia–ischemia insult, with statistical significance in C57BL/6 (*n* = 11), 129SVJ (*n* = 10), CD1 (*n* = 10) and FVB (*n* = 11) LPS-treated animals when compared to the saline-injected controls and to HI alone animals. BALB/c mice (*n* = 10) did not show significantly increased LPS-mediated tissue infarction. Compared with animals not injected prior to hypoxia–ischemia, Saline administration revealed no effect overall (B–I). *p* < 0.05 for Kruskal–Wallis one-way analysis, followed by Dunn multiple comparison test (^∗^for HI + LPS vs. HI + SAL, ^§^for HI + LPS vs. HI alone, ^#^for HI + SAL vs. HI alone, overhead bar for interstrain treatment comparison). Bar scale = 1.2 mm.

The micrographs shown in Fig.1A demonstrate a robust LPS-mediated sensitization to hypoxia–ischemia compared to saline-injected controls. This increase reached significance in the ipsilateral brain hemisphere of four of the five assessed strains (C57BL/6, 129SVJ, CD1, FVB), but not for the BALB/c mice. C57BL/6 (*p* < 0.01), CD1 (*p* < 0.001) and FVB (*p* < 0.01) animals demonstrated the largest increase (Fig.1B, C), with all assessed brain regions significantly affected, including isocortex (Fig.1D): C57BL/6 (*p* < 0.01), CD1 (*p* < 0.001) and FVB (*p* < 0.01); pyriform cortex (Fig.1E): C57BL/6 (*p* < 0.05), CD1 (*p* < 0.01) and FVB (*p* < 0 .01), hippocampus (Fig.1F): C57BL/6 (*p* < 0.01), CD1 (*p* < 0.01) and FVB (*p* < 0.05), striatum (Fig.1G): C57BL/6 (*p* < 0.01), CD1 (*p* < 0.001) and FVB (*p* < 0.01), thalamus (Fig.1H): C57BL/6 (*p* < 0.01), CD1 (*p* < 0.05) and FVB (*p* < 0.05); and external capsule white matter (Fig.1I): C57BL/6 (*p* < 0.01), CD1 (*p* < 0.001) and FVB (*p* < 0.01). 129SVJ animals had a significant increase in tissue infarction in four out of the six individual brain regions (Fig.1D–E, G, I): isocortex (*p* < 0.05), pyriform cortex (*p* < 0.01), striatum (*p* < 0.05) and external capsule (*p* < 0.05), which also resulted in an overall significant increase in brain damage shown in Fig.1B, C (*p* < 0.01).

LPS pre-treated mice also revealed a significant overall increase in tissue infarction when compared to the hypoxia–ischemia alone group in the C57BL/6 (*p* < 0.05), 129SVJ (*p* < 0.01), CD1 (*p* < 0.05) and FVB (*p* < 0.01) strains. This effect was significant in all assessed brain regions across the C57BL/6, 129SVJ, CD1, and FVB strains except for the C57BL/6 thalamus (Fig.1C–I). As with the HI + LPS vs. HI + SAL comparison, there was no significant increase in tissue infarction in LPS-injected BALB/c animals compared to hypoxia–ischemia alone.

#### Infarct volume: treatment and interstrain effects

Overall, of the 35 comparisons possible in Fig.1C–H, i.e. five strains × (six regional + one total tissue loss), there were 27 instances of significant increase for HI + LPS vs. HI + SAL (27/35, highlighted with ^∗^), and same number for the HI + LPS vs. HI alone comparison (27/35, highlighted with ^§^). In each of the five different strains, saline administration alone prior to hypoxia–ischemia injury had minimal or no effect on the overall infarction, for individual regions (Fig.1D–H) as well as for the whole forebrain (Fig.1B): C57BL/6 (*p* = 0.397), 129SVJ (*p* = 0.7019), BALB/c (*p* = 0.2354), CD1 (*p* = 0.6923) and FVB (*p* = 0.4033).

##### HI alone

Total tissue infarction in CD1 (*p* < 0.01) and FVB (*p* < 0.01) strains was significantly larger than in 129SVJ animals (Fig.1C). Regionally (Fig.1D–I), this also applied to the striatum (*p* < 0.05 and *p* < 0.01, respectively), thalamus (*p* < 0.001 and *p* < 0.05) and external capsule (*p* < 0.05 and *p* < 0.01). CD1 animals also had higher tissue loss vs. 129SVJ in the isocortex (*p* < 0.05) and hippocampus (*p* < 0.05).

##### HI + SAL

FVB animals revealed a higher total tissue loss compared to C57BL/6 (*p* < 0.05) and 129SVJ (*p* < 0.01) strains. This was also true for the isocortex (*p* < 0.01 and *p* < 0.01) and striatum (*p* < 0.05 and *p* < 0.01). Compared to just 129SVJ, this also applied to the hippocampus (*p* < 0.05) and external capsule (*p* < 0.05). In the case of CD1 vs. 129SVJ, CD1 animals had significantly more infarction in the striatum and hippocampus (*p* < 0.05).

##### HI + LPS

Total tissue infarction in CD1 and FVB strains was significantly larger than in the C57BL/6 (*p* < 0.01 and *p* < 0.01, respectively), 129SVJ (*p* < 0.01 and *p* < 0.001, respectively) and BALB/c (*p* < 0.05 and *p* < 0.05, respectively). In all assessed regions, FVB again appeared to be the strain with the strongest infarction vs. C57BL/6, 129SVJ, and BALB/c.

##### Interstrain meta-analysis

Across all graphs in Fig.1C–I, significant interstrain differences were only observed between two strain subgroups, in all three treatments (HI alone, HI + SAL, HI + LPS) – with CD1 and FVB in one, and 129SVJ, C57BL/6 and BALB/c in the other subgroup. Generally, the number of significant interstrain differences strongly varied with treatment – HI alone was associated with a total of 10 significant comparisons at 0.05 level out of possible 42 across Fig.1C–I. The number of possible comparisons was calculated as 42 (=2 * 3 * 7), with two standing for the number of subgroup A strains, three for the number of subgroup B strains and seven for the number of different brain regions tested (including the total brain volume loss). HI + SAL was associated with 11 out of 42 and HI + LPS with 26 out of 42. In other words, in the case of brain tissue volume loss, the overall number of significant strain differences did not change with saline pre-treatment, but increased with LPS sensitization compared against HI alone (*p* < 0.001 in Chi-Square test in a 2 × 3 matrix, followed by the individual 2 × 2 comparison), as well as against HI + SAL (*p* < 0.001).

In terms of 129SVJ, C57BL/6 and BALB/c, compared with subgroup B strains, there were 30 significant differences at 0.05 level (out of 42 possible) for 129SVJ, 12/42 for C57BL/6 and 5/42 for BALB/c. Overall, 129SVJ displayed a significantly higher number of differences vs. FVB and CD1 than for C57BL/6 or BALB/c (*p* < 0.001 for either, as above). Differences for C57BL/6 vs. BALB/c did not reach significance level (*p* = 0.057 in Chi-Square test). In terms of CD1 and FVB, compared with subgroup A strains, there were 22 significant differences (out of 63 possible) for CD1 and 25/63 for FVB, these differences were not significantly different in Chi-Square test (*p* = 58%). Generally, in terms of extent of tissue loss, current data meta-analysis for extent of brain volume loss would thus suggest HI < HI + SAL << HI + LPS for treatment, and 129SVJ < C57BL/6 << BALB/c << CD1 < FVB for the affected mouse strain.

### Injury score

**Fig. 2:**
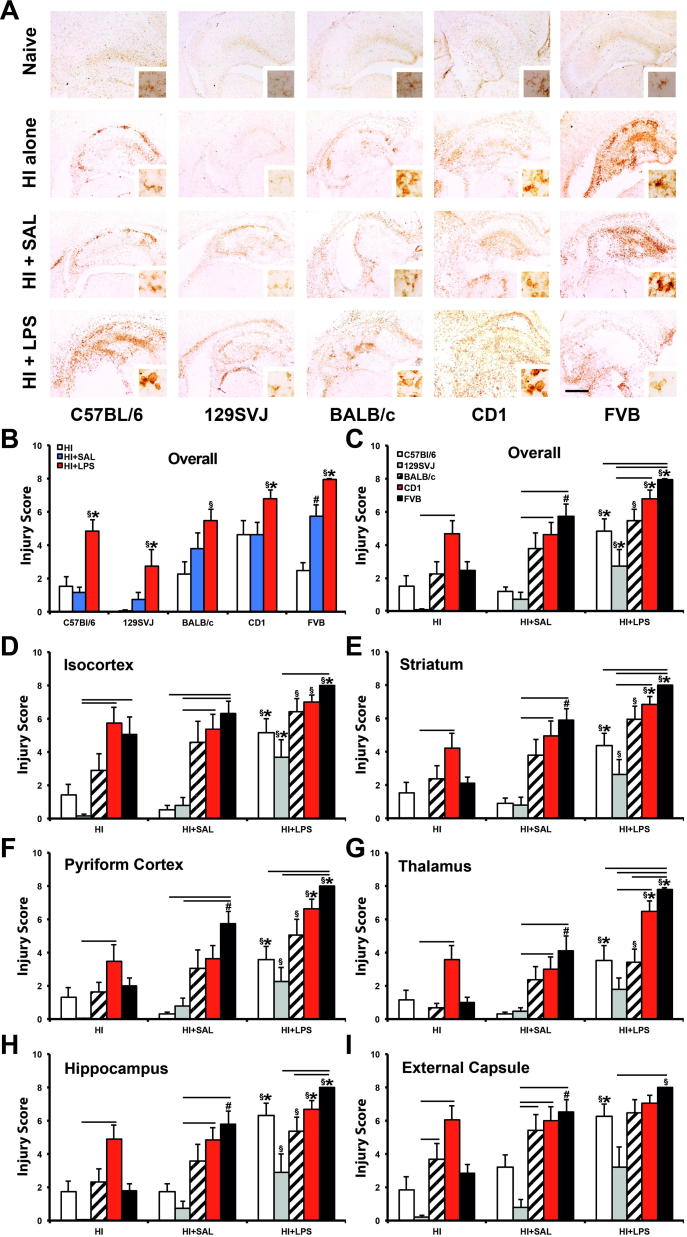
LPS sensitization to neonatal hypoxia–ischemia substantially increases microglial activation and neuronal cell loss on the C57BL/6, 129SVJ, CD1 and FVB background. (A) Microglial activation staining (alphaM) of coronal hippocampal sections of C57BL/6, 129SVJ, BALB/c, CD1 and FVB strains 48 h following hypoxia–ischemia. The upper row shows untreated naïve animals, second row shows mice exposed to hypoxic–ischemic insult alone (HI alone), third row those injected 12 h earlier with saline (HI + SAL), and lower row animals pre-treated with 0.6 μg LPS/g body weight (HI + LPS). Nissl staining used for neuronal cell loss scoring is shown in Fig. 1. Note the lack of microglial activation in the naïve group. The LPS-treated BALB/c strain showed minimal increase in microglial activation and neuronal cell loss, when compared to saline-treated controls. However, comparison with HI alone animals revealed a significant increase in combined injury score. Compared with animals not injected prior to hypoxia–ischemia, Saline administration elicited an increase in injury score overall (B, C) as well as in most FVB ipsilateral brain regions (D–I). Interstrain treatment comparison revealed that both CD1 and FVB background strains had the highest levels of combined injury score, with significance being reached when compared to 129SVJ animals (C–I). *p* < 0.05 for Kruskal–Wallis one-way analysis, followed by Dunn multiple comparison test (^∗^for HI + LPS vs. HI + SAL, ^§^for HI + LPS vs. HI alone, ^#^for HI + SAL vs. HI alone, overhead bar for interstrain treatment comparison). Bar scale = 0.5 mm.

In addition to tissue loss, LPS pre-treatment is known to significantly increase ipsilateral microglial activation and neuronal cell loss, which are scored blindly and then combined in the brain injury score (Kendall et al., 2011). Fig.2A gives an overview of the effects of LPS sensitization on microglial activation following hypoxic–ischemic insult in the current study, the effects on the brain injury score are shown in Fig.2B–I. Compared with the saline controls, the highest overall increase following LPS sensitization was observed in the C57BL/6 background, where all assessed brain regions (Fig.2D–I) as well as their overall average (Fig.2B, C) showed a significant increase in injury score: isocortex (*p* < 0.001), pyriform cortex (*p* < 0.01), hippocampus (*p* < 0.01), striatum (*p* < 0.05), thalamus (*p* < 0.05) and external capsule (*p* < 0.05) (Fig.2D–I). FVB animals were the second most affected, with a significant increase in all regions except external capsule: isocortex (*p* < 0.05), pyriform cortex (*p* < 0.05), hippocampus (*p* < 0.05), striatum (*p* < 0.05) and thalamus (*p* < 0.05) (Fig.2D–H). In CD1 animals the pyriform cortex (*p* < 0.05), hippocampus (*p* < 0.05), striatum (*p* < 0.05) and thalamus (*p* < 0.05) were also significantly affected (Fig.2E–H), 129SVJ LPS-treated animals only showed a substantial increase (*p* < 0.05) in the isocortex (Fig.2B).

#### Brain injury score: treatment and interstrain meta-analysis

Unlike the tissue loss in Fig.1, the injury score comparison between HI + LPS and HI alone revealed a more extensive effect than that observed for HI + LPS vs. HI + SAL. Significant increases (*p* < 0.05) were now also present for the BALB/c strain (Fig.2C–H), with the external capsule being the only exception (Fig.2I).

Overall, there were 31 instances (out of 35 possible) of significant increase for HI + LPS vs. HI alone, compared to 20/35 for HI + LPS vs. HI + SAL comparison (*p* < 0.01 in Chi-Square test). BALB/c effects (*n* = 6) contributed to roughly half of the increase in the overall number of significant changes for HI + LPS vs. HI alone (*n* = 11). In a similar vein, the FVB strain also showed a significant, saline injection-associated increase in injury score compared with hypoxia–ischemia alone across the entire ipsilateral forebrain hemisphere. This effect was observed overall (Fig.2C), but also regionally, in pyriform cortex, hippocampus, striatum and external capsule (Fig.2E–G, I). Generally, there was little evidence of brain injury in the contralateral hemisphere in any of the five strains, irrespective of the type of insult (data not shown).

As with tissue volume loss, with the exception of the BALB/c external capsule (Fig.2I), significant interstrain differences for injury score were only observed between the two strain subgroups, in all three treatments (HI alone, HI + SAL, HI + LPS) – with CD1 and FVB in one, and 129SVJ, C57BL/6 and BALB/c in the other subgroup. In the B strain subgroup, there was again no significant difference in the number of significant comparisons for CD1 (16/63) and for FVB (23/63). In the A subgroup, 129SVJ displayed a significantly higher number of differences vs. FVB and CD1 (31/42), than for C57BL/6 (6/42) or BALB/c (2/42) (*p* < 0.001 for either, as above). Differences for C57BL/6 vs. BALB/c did not reach significance level (*p* = 0.137).

In terms of treatment – HI alone was associated with a total of eight significant comparisons at 0.05 level out of possible 42 across Fig.1C–I. HI + SAL with was associated with 15 out of 42 and HI + LPS with 16 out of 42. Data suggested that pre-treatment per se would be associated with a significantly higher number of strong differences between strains, but this did not reach significance (*p* = 0.120 in the initial Chi-Square test in a 2 × 3 matrix).

### TUNEL+ cells

**Fig. 3:**
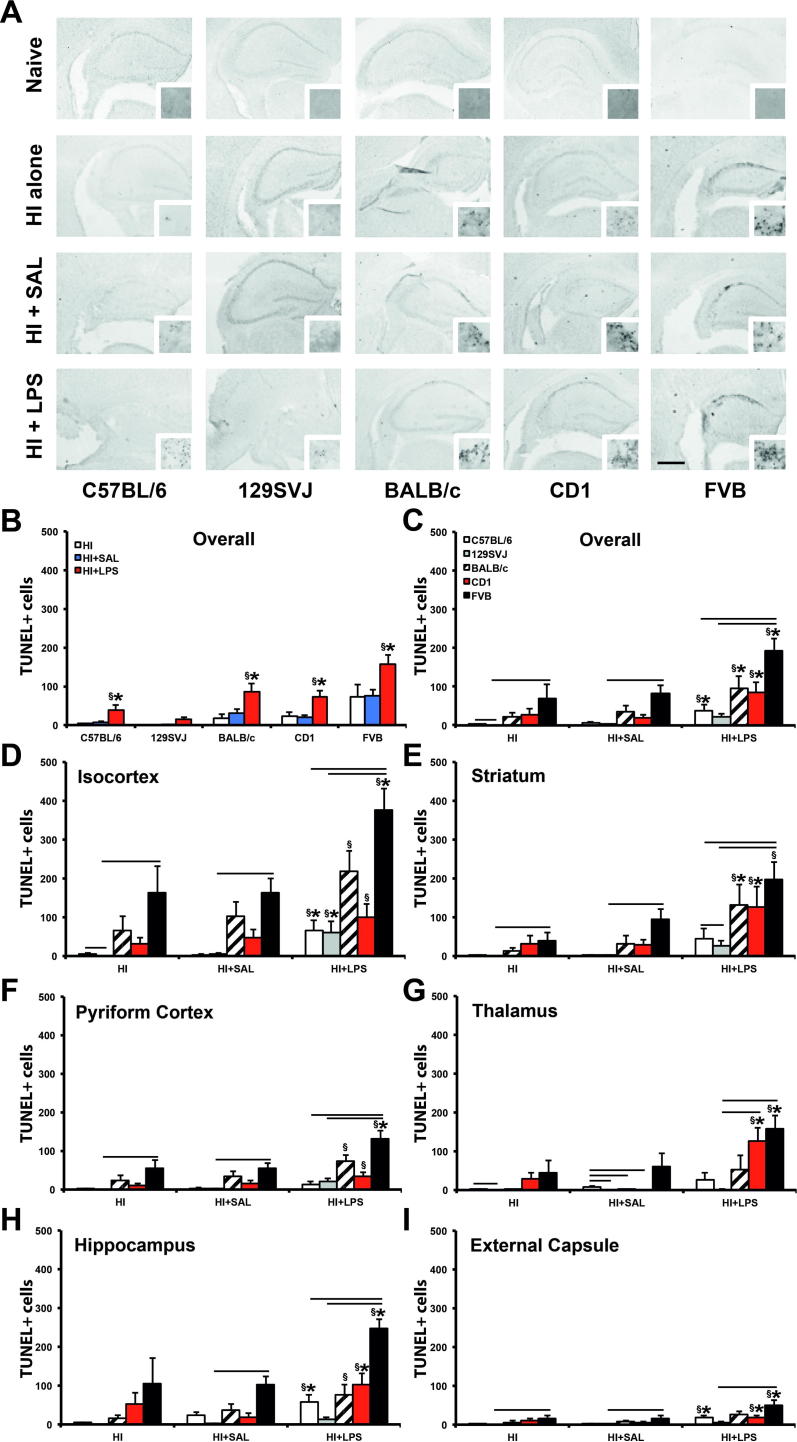
DNA fragmentation-associated cell death detected with TUNEL is increased in animals pre-treated with LPS across all assessed mouse strains. (A) TUNEL assay of 40-μm coronal hippocampal sections of C57BL/6, 129SVJ, BALB/c, CD1 and FVB strains 48 h following hypoxia–ischemia. The upper row shows untreated naïve animals, second row mice exposed to hypoxic–ischemic insult alone (HI alone), third row those injected 12 h earlier with saline (HI + SAL), and lower row animals pre-treated with 0.6 μg LPS/g body weight (HI + LPS). Saline administration prior to hypoxia–ischemia had no significant impact, whereas LPS pre-treatment resulted in substantial increase in TUNEL+ cell death. (B, C) Overall effect across all six brain regions, both in terms of strain (B) and treatment (C) comparison. (D–I) Significantly increased LPS-induced cell death was also present in 5/6 regions in the FVB, 3/6 in the C57BL/6 and CD1, and in 2/6 brain regions of the BALB/c animals, when compared to saline-treated controls. Comparison with HI alone demonstrated an even higher LPS-mediated increase in TUNEL+ cell counts. Despite 129SVJ animals showing no overall significant increase in cell death (A), the isocortex was still significantly affected when comparing HI + LPS vs. HI + SAL and HI + LPS vs. HI alone (D). Interstrain comparison revealed that FVB was the most significantly affected strain, especially in comparison to 129SVJ mice followed by CD1 animals. *p* < 0.05 for Kruskal–Wallis one-way analysis, followed by Dunn multiple comparison test (^∗^for HI + LPS vs. HI + SAL, ^§^for HI + LPS vs. HI alone, ^#^for HI + SAL vs. HI alone, overhead bar for interstrain treatment comparison). Bar scale = 0.5 mm.

Results in Fig. 1, Fig. 1 demonstrate that LPS administration prior to hypoxic–ischemic insult has a significant detrimental effect within the first 48 h. Hypoxia–ischemia is associated with a rapid increase in neural cell death- as shown in Fig.3A; this is also confirmed by TUNEL histochemistry for nuclear fragmentation associated with hypoxia–ischemia-induced cell death at 48 h post-insult. Quantification of this DNA fragmentation on ipsilateral side in Fig.3B–I revealed that LPS pre-treatment led to a substantial overall increase in the number of TUNEL+ cells in the ipsilateral hemisphere of C57BL/6 (*p* < 0.05), BALB/c (*p* < 0.05), CD1 (*p* < 0.05) and FVB (*p* < 0.05) background strains when compared to saline-treated controls (Fig.3B, C). TUNEL+ cell counts across the different individual brain regions revealed the highest increase in TUNEL+ cells in the isocortex of C57BL6 (*p* < 0.05), 129SVJ (*p* < 0.05) and FVB (*p* < 0.01) animals (Fig.3D), hippocampus of C57BL/6 (*p* < 0.01), CD1 (*p* < 0.05) and FVB (*p* < 0.001) mice (Fig.3H) and external capsule of C57BL/6 (*p* < 0.01), BALB/c (*p* < 0.05) and FVB (*p* < 0.05) pups (Fig.3I). This was followed by the striatum of BALB/c (*p* < 0.05) and CD1 (*p* < 0.05) animals (Fig.3E) and thalamus in CD1 (*p* < 0.05) and FVB (*p* < 0.05) mice (Fig.3G). Pyriform cortex was the least affected forebrain region, showing only a significant increase in cell death in the FVB (*p* < 0.01) background strain (Fig.3F).

#### TUNEL+ cell density: treatment and interstrain meta-analysis

The TUNEL+ density comparison between HI + LPS and HI alone in Fig.3C–I revealed 24 instances (out of 35 possible) of a significant increase for HI + LPS vs. HI alone, compared to 18/35 for HI + LPS vs. HI + SAL comparison. These differences did not reach statistical significance in a Chi-Square test (*p* = 0.143). There was also no significant, saline injection-associated increase in density of TUNEL+ dying cells compared with hypoxia–ischemia alone across the entire ipsilateral forebrain hemisphere, in any of the studied subregions.

As with tissue volume loss, with three exceptions (Fig.3E, G, I), significant interstrain differences for TUNEL+ density were only observed between the two strain subgroups, in all three treatments (HI alone, HI + SAL, HI + LPS) – with CD1 and FVB in one, and 129SVJ, C57BL/6 and BALB/c in the other subgroup. In the B strain subgroup, there was a highly significant difference in the number of strong inter-subgroup differences for CD1 (1/63) and for FVB (23/63). In the A subgroup, 129SVJ again displayed a significantly higher number of inter-subgroup differences vs. FVB and CD1 (19/42), than for C57BL/6 (5/42) or BALB/c (0/42) (*p* < 0.001 for either, as above). Moreover, differences for C57BL/6 vs. BALB/c also reach significance level (*p* = 0.021).

In terms of treatment – HI alone was associated with a total of 5 significant interstrain comparisons at 0.05 level out of possible 42 across Fig.1C–I. HI + SAL was associated with six out of 42 and HI + LPS with 13 out of 42. As in Fig.1, the data suggested that treatment with LPS is associated with a higher number of strong differences in TUNEL+ density between strains, but in the current case this just barely missed significance level (*p* = 0.053 in the initial Chi-Square test in a 2 × 3 matrix, *p* = 0.068 for the post hoc 2 × 2 comparison of HI + LPS vs. HI + SAL).

### GFAP immunoreactivity occluded side

**Fig. 4:**
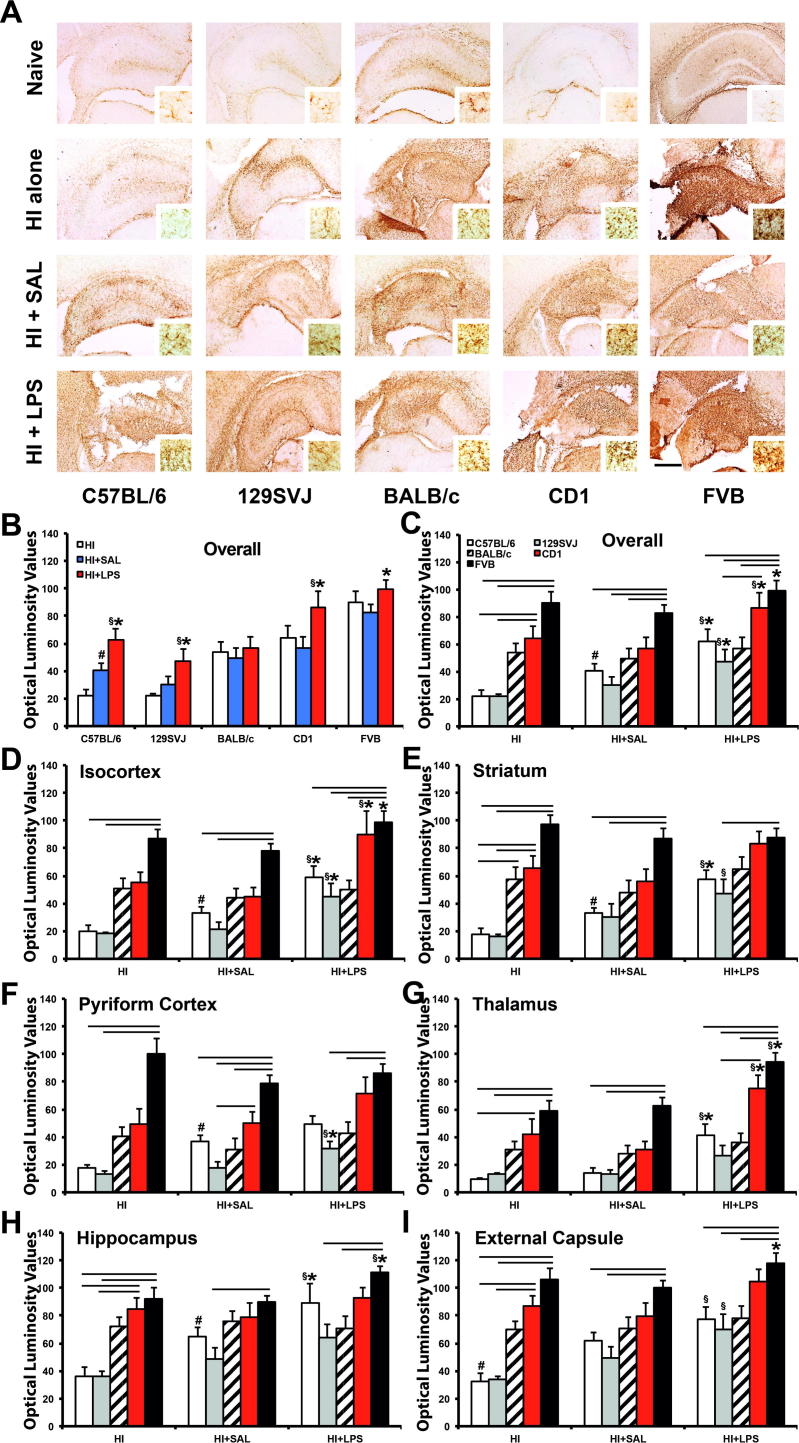
LPS-mediated sensitization to neonatal hypoxia–ischemia induces astrogliosis with increased GFAP-immunoreactivity (GFAP-IR) in the forebrain hemisphere on the side with carotid occlusion. (A) Hippocampal sections showing distribution of GFAP-IR in C57BL/6, 129SVJ, BALB/c, CD1 and FVB naïve animals (top row), only exposed to HI insult (second row), previously injected with saline (third row) or pre-exposed to LPS 12 prior to hypoxia–ischemia (last row). Note the clear increase in GFAP-IR the dorsoparietal isocortex, rostral hippocampus, striatum and external capsule in the saline pre-injected C57BL/6 animal (A). (B, C) Overall GFAP-IR per strain (B) and per treatment (C). (D–I) Quantification of astrogliosis using OPTIMAS-assessment of GFAP-IR with optical luminosity values (OLV) showed a significant, LPS-induced increase in the C57BL/6, 129SVJ, CD1 and FVB strains. C57BL/6 and FVB animals appeared most affected, overall (B, C) and with 4/6 regions showing a significant increase (D–I). This effect was also seen in 3/6 CD1 and 2/6 129SVJ brain regions, but not in the BALB/c strain. Interstrain comparison demonstrated a clear significant astroglial immunoreactivity for the FVB strain when compared to most strains in both HI + SAL and HI + LPS treatments, and particularly with C57BL/6 and 129SVJ in terms of HI alone injury. *p* < 0.05 for Kruskal–Wallis one-way analysis, followed by Dunn multiple comparison test (^∗^for HI + LPS vs. HI + SAL, ^§^for HI + LPS vs. HI alone, ^#^for HI + SAL vs. HI alone, overhead bar for interstrain treatment comparison). Bar scale = 0.5 mm.

**Fig. 5:**
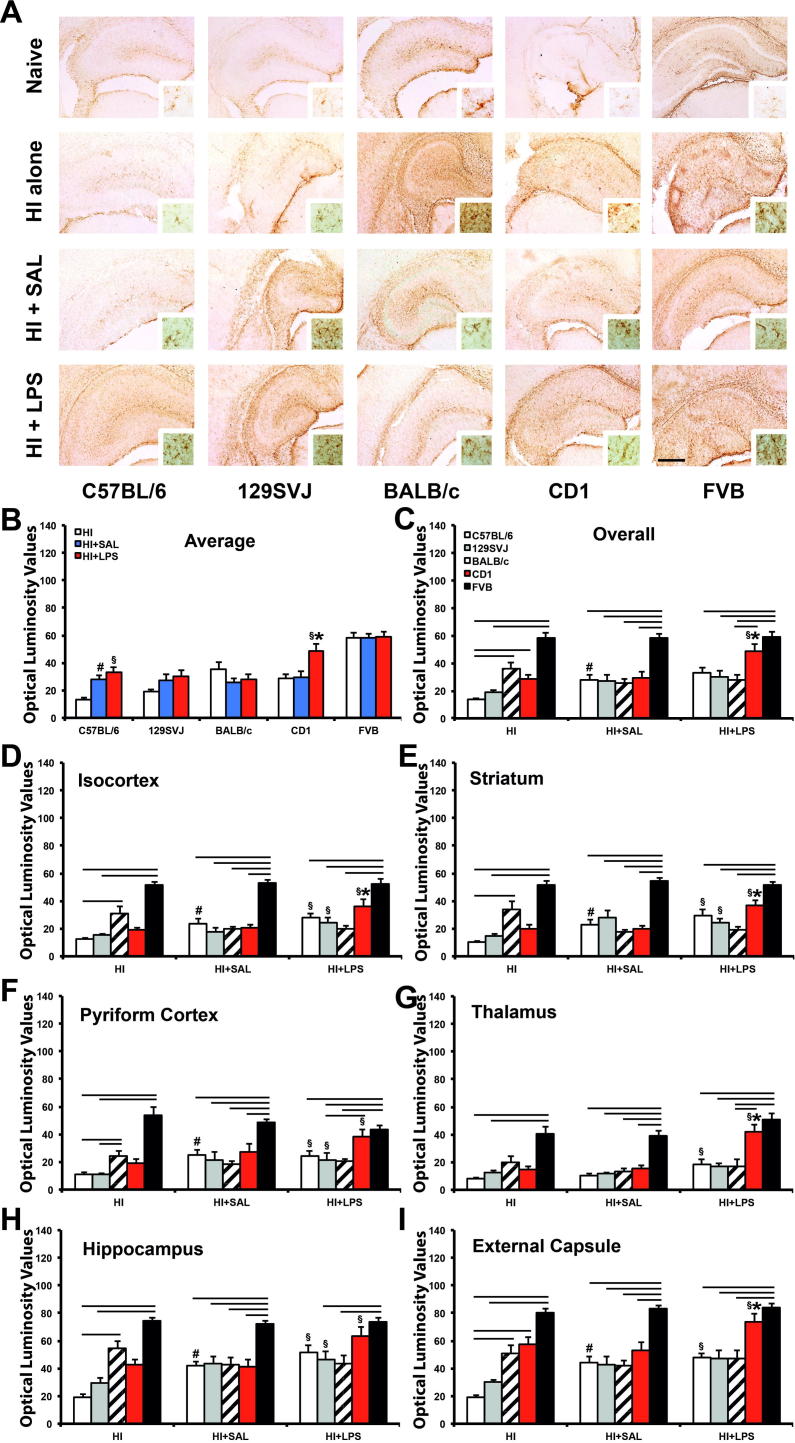
Strain-specific appearance of contralateral astrogliosis in the non-occluded forebrain hemisphere following hypoxia–ischemia. (A) Hippocampal sections showing distribution of GFAP-IR in C57BL/6, 129SVJ, BALB/c, CD1 and FVB naïve animals (top row), only exposed to HI insult (second row), previously injected with saline (third row) or pre-exposed to LPS 12 prior to hypoxia–ischemia (last row). Distribution of GFAP-IR in C57BL/6 animals shows an increase in contralateral astroglial activation in the HI + SAL group when compared to HI alone. This increase was sometimes more pronounced in LPS-treated mice (B, C). (D–I) OPTIMAS Quantification of GFAP-IR showed significant treatment-induced increase in C5BL/6 and CD1 strains, in the former for HI + SAL vs. HI alone and in latter for HI + LPS vs. HI + SAL and HI alone, both overall (B, C) as well as in 5/6 and 4/6 brain regions, respectively (D–I). Interestingly, interstrain comparison revealed substantially higher GFAP-IR in FVB animals when compared to C57BL/6 and 129SVJ mice across all three treatments – HI alone, HI + SAL, HI + LPS – however, FVB animals did not show a difference in contralateral GFAP-IR between the three treatments. *p* < 0.05 for Kruskal–Wallis one-way analysis, followed by Dunn multiple comparison test (^∗^for HI + LPS vs. HI + SAL, ^§^for HI + LPS vs. HI alone, ^#^for HI + SAL vs. HI alone, overhead bar for interstrain treatment comparison). Bar scale = 0.5 mm.

Astroglial activation following hypoxic–ischemic insult, and pre-sensitization with LPS and to lesser extent with saline, is associated with a strong increase in GFAP immunoreactivity (GFAP-IR) shown in Fig. 4, Fig. 5A, focusing on ipsilateral and contralateral hippocampus, respectively, and surrounding brain regions. Quantification of this GFAP-IR with OPTIMAS using optical luminosity value (OLV) system (Fig. 4, Fig. 5B–I) revealed that LPS-mediated sensitization significantly increased astrocytic activation compared to the saline-injected controls.

On the occluded side (Fig.4C–D), the effect was present in four out of five strains, with the exception of BALB/c animals (Fig. 4, Fig. 5). The C57BL/6 (*p* < 0.05) and FVB (*p* < 0.05) strains were particularly affected, with a significant impact not just overall (Fig.4B, C), but also in four out of six regions, including isocortex (*p* < 0.05 and *p* < 0.05, respectively), hippocampus (*p* < 0.05 and *p* < 0.01, respectively) and thalamus (*p* < 0.01 and *p* < 0.01, respectively). The ipsilateral striatum was also significantly affected in the C57BL/6 strain (*p* < 0.05), whereas FVB animals were also particularly affected in the external capsule (*p* < 0.05) (Fig.4E–J). Overall strong effects were also observed in the 129SVJ (*p* < 0.05) and CD1 (*p* < 0.01) strains (Fig.4B, C), but on the regional level these were only significant in the isocortex (*p* < 0.01), striatum (*p* < 0.05) and thalamus (*p* < 0.01) of CD1 mice (Fig.4E, H–I) and isocortex (*p* < 0.05) and pyriform cortex (*p* < 0.05) of 129SVJ animals (Fig.4E–F).

Of all five assessed strains, C57BL/6 animals also demonstrated that vehicle/saline administration followed by hypoxia–ischemia increased ipsilateral GFAP-IR (Fig.4A–C), compared to the untreated hypoxia–ischemia controls in this strain (*p* < 0.01). This effect was significant overall (Fig.4B, C) and in all subregions except the thalamus (Fig.4G), i.e. in the isocortex (*p* < 0.05), pyriform cortex (*p* < 0.01), hippocampus (*p* < 0.05), striatum (*p* < 0.01) and external capsule (*p* < 0.01) regions (Fig.4E–H, J). 129SVJ (*p* = 0.462), BALB/c (*p* = 0.5134), CD1 (*p* = 0.616) and FVB (*p* = 0.3233) backgrounds did not show this ipsilateral effect.

#### Ipsilateral GFAP-IR: treatment and interstrain meta-analysis

Comparison of ipsilateral GFAP-IR between HI + LPS and HI alone, and HI + LPS vs. HI + SAL in Fig.4C–I revealed virtually identical results, with 16 instances (out of 35 possible) of a significant increase for HI + LPS vs. HI alone, and 17/35 for the HI + LPS vs. HI + SAL comparison (*p* = 0.811 in Chi-Square test).

143). With the exception of the striatum brain region in BALB/c animals HI-treated (Fig.4E), significant interstrain differences for ipsilateral GFAP-IR were again only observed between the two strain subgroups, in all three treatments (HI alone, HI + SAL, HI + LPS) – with CD1 and FVB in one, and 129SVJ, C57BL/6 and BALB/c in the other subgroup. In the B strain subgroup, there was a highly significant difference in the number of significant inter-subgroup comparisons for CD1 (12/63) and for FVB (46/63). In the A subgroup, 129SVJ and C57BL/6 displayed a significantly higher number of inter-subgroup differences vs. FVB and CD1 (28/42 and 22/42, respectively), than BALB/c (8/42) (*p* < 0.002 for either, as above). Differences for 129SVJ vs. C57BL/6 vs. BALB/c in terms of GFAP-IR did not reach significance level (*p* = 0.182).

In terms of treatment – HI alone was associated with a total of 23 significant interstrain comparisons at 0.05 level out of possible 42 across Fig.1C–I. HI + SAL with was similarly associated with 16 out of 42 and HI + LPS with 19 out of 42. The overall results are fairly similar (*p* = 0.307 in the initial Chi-Square test in a 2 × 3 matrix), excluding a specific effect of injecting LPS or saline on significantly increasing ipsilateral interstrain differences for GFAP-IR.

### GFAP immunoreactivity contralateral, non-occluded side

Most of the significant changes observed on the occluded side were also present in the contralateral, non-occluded forebrain hemisphere. In particular, a similar significant increase in GFAP-IR following saline injection was also observed on the non-occluded, contralateral side of the C57BL/6 strain (Fig.5A–C). On the regional level (Fig.5D–F, H–I), a significant increase following saline injection was also observed in the isocortex (*p* < 0.001), pyriform cortex (*p* < 0.01), hippocampus (*p* < 0.001), striatum (*p* < 0.001) and external capsule (*p* < 0.001). Interestingly, the discrete neuroanatomical localization of increased GFAP-IR in the contralateral hemisphere (shown in Fig.5A) was only present in the mid-lateral part of isocortex, pyriform cortex, hippocampus, striatum and external capsule. This appeared to resemble the distribution of the affected, mostly medial cerebral artery (MCA) territory on the occluded, ipsilateral side (Fig.4A). Animals from the other four strains, i.e. 129SVJ (*p* = 0.549), BALB/c (*p* = 0.1128), CD1 (*p* = 0.7871) and FVB (*p* = 0.936) animals did not show contralateral difference between the HI alone and HI + SAL littermates (Fig.5B, C).

#### Contralateral GFAP-IR: treatment and interstrain meta-analysis

Unlike the ipsilateral side, contralateral GFAP-IR comparisons between HI + LPS and HI alone, and HI + LPS vs. HI + SAL in Fig.5C–I revealed highly disparate results, with 17 instances (out of 35 possible) of a significant increase of interstrain differences for HI + LPS vs. HI alone, vs. just 5/35 for the HI + LPS vs. HI + saline comparisons (*p* < 0.01 in Chi-Square test).

Unlike Fig. 1, Fig. 2, Fig. 3, Fig. 4, the data for BALB/c and CD1 in Fig.5 also showed more frequent exceptions in terms of significant intra-group differences. These were fairly numerous, with 7/42 for BALB/c, and 7/21 for CD1, respectively. Thus, these strains no longer strictly conformed to subgroup A and B. If only strain differences between the predefined subgroups were assessed, there was however a highly significant difference for the B subgroup in the number of significant inter-subgroup comparisons for CD1 (5/63) and for FVB (55/63), with a *p* < 0.01 in Chi-Square test. In the A subgroup, all three strains – 129SVJ, C57BL/6 and BALB/c displayed a roughly similar number of inter-subgroup differences vs. FVB and CD1 (22/42, 22/42, and 15/42, respectively), with a *p*-value of 0.210 for the initial Chi-Square test in a 2 × 3 matrix.

In terms of treatment – HI alone was associated with a total of 16 significant interstrain comparisons at 0.05 level out of possible 42 across Fig.1C–I. HI + SAL with was similarly associated with 21/42 and HI + LPS with 23/42. The overall ratio of results are fairly similar (*p* = 0.331 in the initial Chi-Square test in a 2 × 3 matrix), arguing against a specific effect of injecting LPS or saline on significantly increasing interstrain differences for contralateral GFAP-IR.

## Discussion

### Mouse strain and the extent of hypoxic–ischemic damage

The current study shows the strain background influencing the response to neonatal hypoxic–ischemic insult in the absence and presence of LPS, the extent of tissue volume loss and neural cell death and the level of injury-associated inflammatory markers. Strain-based susceptibility to hypoxia–ischemia was particularly low in the 129SVJ and then in the C57BL/6 inbred strains. BALB/c mice showed moderate, and CD1 and FVB animals a strong response, which was frequently particularly pronounced in the case of FVB. Interestingly, in the case of GFAP-IR, higher levels were already observed in the naïve controls in the FVB strain, and thus could contribute to higher GFAP-IR levels in the HI alone group of this strain. We acknowledge that the precise extent of the changes in GFAP-IR from naïve to HI alone in different strains has not been specifically addressed here and should be assessed in a future study with naïve animals serving as baseline.

For the purpose of the current study, the levels in the HI alone group were set as the baseline for comparison between strains, and for comparison with LPS sensitization or saline injection, as a key purpose here was to compare response in these two different sets of treatment. LPS pre-treatment sensitized all five strains to hypoxia–ischemia but to a different extent: FVB animals showing most overall damage and injury marker response, followed by CD1, C57BL/6 and 129SVJ. BALB/c strain responded weakest to LPS – a significant effect versus saline pre-injected controls was only observed in some brain subregions for TUNEL+ cell death (Fig.3). Against the somewhat lower levels of HI only littermates the LPS-pre-treated BALB/c animals also showed significance for brain injury score (Fig.2), but even here, there was no significant HI + LPS/HI alone effect for tissue volume loss (Fig.1) or for ipsi- (Fig.4) and contralateral (Fig.5) astrogliosis. C57BL/6 and FVB strains had a discrete but significant response with pre-sensitization to injection with saline, and C57BL/6, 129SVJ and CD1 mice also revealed pronounced but localized contralateral astrogliosis.

Previous adult ischemia studies have demonstrated cerebrovascular architecture variations between mouse strains (Barone et al., 1993, Fujii et al., 1997, Maeda et al., 1999, Beckmann, 2000). C57BL/6 mice have an incompletely formed circle of Willis (Fujii et al., 1997, Maeda et al., 1998) and a larger MCA territory supply than 129SVJ mice (Connolly et al., 1996), which can partially explain observations of a larger brain infarct following artery occlusion in the C57BL/6 strain (Connolly et al., 1996). A study using unilateral carotid ligation in postnatal day 12 CD1 and C57BL/6 mice also revealed that the C57BL/6 background was less vulnerable both to brain injury and seizures than the CD1 strain (Comi et al., 2009). The acute hypoxia study in adult C57BL/6 and CD1 mice also demonstrated a higher tolerance and survival in the C57BL/6 inbred strain (Zwemer et al., 2007). To our knowledge the only other study to have looked at the effects of neonatal hypoxia–ischemia following unilateral carotid ligation and 30 min hypoxia in multiple mouse strains (Sheldon et al., 1998) revealed minimal brain injury in 129SVJ mice, moderate damage in C57BL/6 and a particularly strong response in the CD1 strain.

All these results are generally corroborated in our findings. Simply judging by average results, 129SVJ showed the least damage, followed by C57BL/6, then BALB/C and CD1, with FVB being, overall, the most-affected. However, almost all significant interstrain differences – with few exceptions – with 129SVJ, C57BL/6 and BALB/c in one, and CD1 and FVB in the other subgroup. This fragmentation into these two subgroups also continued across all three treatments (HI alone, HI + SAL, HI + LPS).

Generally, the ability to pick up a significant difference is a function of the size of the underlying effect, intra-group variability and sample size. Strong effects will be picked up directly in direct comparisons even with moderate sample size, but more moderate effects can also have an impact on more or less frequent detection of significant differences against other, strongly disparate strains. In the current study, different strains within each subgroup also showed clear differences using the Chi-Square test, in the number of significant comparisons with strains of the opposite subgroup for tissue-loss and most damage-associated markers. Strains within subgroup A (129SVJ, C5BL/6, BALB/c) exhibited significant differences for loss of brain volume, brain injury index, density of TUNEL+ cell death and ipsilateral GFAP-IR, strains within subgroup B (CD1, FVB) for TUNEL and ipsi- and contralateral GFAP-IR. Inside each subgroup, 129SvJ and FVB occupied the more extreme position, which resulted in more significant comparisons with strains of the opposite group.

Overall, these positions continued across all three treatments (HI alone, HI + SAL, HI + LPS), different forebrain sub-regions and markers of brain damage. Moreover, the same also held true with the more general fragmentation into the two subgroups. This could suggest the presence of generic low and high responder backgrounds, although at present we cannot exclude that this apparent sub-grouping is a random feature that arose by chance, and there is a continuum of possible responses. It will be interesting to see if there are biochemical markers very early after neonatal hypoxia–ischemia or in naïve postnatal day 7 pups that differ between strains and whether they show the same alignment into these two strain subgroups, and possibly, the same interstrain fragmentation.

### Saline-stressor effect in the different strain backgrounds

This study revealed a saline-mediated increased response in ipsilateral combined injury score of FVB mice. The C57BL/6 strain showed enhanced astrogliosis on both occluded and non-occluded hemispheres in the saline group. The saline used in our experimental design was obtained from veterinary sources as sterile intravenous infusion, arguing against endotoxin contamination or infection, and instead pointing to other, stress-associated factors.

Stressful events can cause short and/or long-term behavioral and neurochemical adverse outcomes and influence circulating and brain cytokine activity (Gibb et al., 2008, Audet et al., 2010). Individual animals as well as different strains show diverse sensitivity to stress, where BALB/c mice are highly stress reactive in comparison to C57BL/6 (Anisman et al., 2008, Anisman et al., 2001). Social stress alone (plus saline), or in combination with acute immune challenges increases production of plasma corticosterone in both strains. Additionally, vehicle-treated C57BL/6 mice have higher interleukin-6 (IL-6) expression than the corresponding BALB/c group, suggesting strain-associated response to stress (Gibb et al., 2011). Similarly, forced-swim stressor alone (plus vehicle) significantly raised corticosterone levels in both strains (Connor et al., 1997).

Although LPS-mediated up-regulation of IL-1β and tumor necrosis factor alpha (TNFα) was observed in both strains, it was more evident in BALB/c animals. Interestingly, the presence of swim-stressor attenuated IL-1β and TNFα up-regulation in the BALB/c mice, suggesting altered immune activation in BALB/c animals when compared to the more normo-sensitive C57BL/6 (Browne et al., 2012). These findings suggest that in our experimental design the stress of dam separation and intraperitoneal administration of saline could affect the immune response, as seen in the assessment of immune-associated markers for astrogliosis and microglial activation of C57BL/6 and FVB strains respectively. Interestingly, there was also a non-significant increase in brain damage in saline-treated BALB/c animals when compared to hypoxia–ischemia alone mice, again suggesting a moderate stress-associated effector for this strain.

### Strain impact on combined inflammation and hypoxic–ischemic insults

Our study demonstrates strong LPS-mediated sensitization to neonatal hypoxia–ischemia in the C57BL/6, 129SVJ, CD1 and FVB strains. However, this degree of sensitization varies, and the BALB/c strain produced a reduced response. We have used one single set of dose and timing only in this study, whereas other studies have used different protocols such as LPS dose of 0.3 mg/kg 12 h prior to insult (Wang et al., 2009). Therefore it is possible that the BALB/c response to LPS would differ with different doses and/or time courses. It is also possible that an increase in *n* numbers may reduce variability associated with this model, and improve significance.

Hypoxia–ischemia triggers inflammatory response and cytokine production. Pre-treatment of CD1 mice with TNFα monoclonal antibody significantly reduces infarction following MCA occlusion (Yang et al., 1998). BALB/c mice pre-treated with TNF receptor linked to polyethylene glycol have smaller infarct compared to controls (Nawashiro et al., 1997). These data suggest strain-independent modulatory effect of TNFα on ischemic-induced brain damage. However, microglial synthesis of TNFα within the focal and border zone of cerebral infarction is strain dependent. BALB/c mice have larger infarction with significantly fewer TNFα-producing microglia in comparison to C57BL/6 animals (Lambertsen et al., 2002).

Although the additional extent of volume loss or astrogliosis never reached significance in BALB/c animals pre-treated with LPS, our data do show LPS-induced effects for TUNEL+ cell death. In addition, compared to HI alone as a surrogate control, they also show effects for the brain injury score. If a saline-associated stressor is considered for this strain, and therefore a comparison between LPS-treated and hypoxia–ischemia alone treatments is performed, one can see that LPS pre-sensitization then becomes significant, increasing combined microglial activation and neuronal cell loss as well as TUNEL+ cell death assessments.

Our results show that hypoxia–ischemia has a minimal response effect on 129SVJ animals. Despite a substantial increase with LPS pre-treatment, the response to the synergistic model was generally smaller when compared to the other strains. Besides cerebrovascular anatomical differences (Fujii et al., 1997, Maeda et al., 1998, Beckmann, 2000), studies looking at the 129SVJ strain immune profile, have shown defective inflammatory cell recruitment (White et al., 2002, Hoover-Plow et al., 2008). C57BL/6 mice exhibit the strongest response in total cell recruitment following intraperitoneal administration of thioglycollate, followed by BALB/c, CD1 and lastly 129SVJ mice (White et al., 2002). Inflammatory stimulus in C57BL/6 and 129SVJ animals revealed that 129SVJ had a lower number of recruited leucocytes and macrophages (Hoover-Plow et al., 2008). This reduced 129SVJ ability to recruit macrophages was also shown in a model of *Mycobacterium tuberculosis*, where 129SVJ mice were significantly more susceptible than C57BL/6 animals (Medina and North, 1998). Our data demonstrate diminished response to hypoxia–ischemia in the 129SVJ animals. Despite successful sensitization with LPS, the 129SVJ immune response and brain damage was still smaller than most of the other strains, suggesting background-dependent alteration of immune cell recruitment might be responsible for the 129SVJ strain resistance to hypoxia–ischemia.

### Hypoxia–ischemia-induced astrogliosis

In addition to increased GFAP-IR in the ipsilateral hemisphere of the 4 strains strongly responding to LPS, two of these strains – C57BL/6 and CD1 - also showed a significant contralateral response, which followed the ipsilateral change. C57BL/6 animals had a substantial increase in astroglial immunoreactivity in the saline-treated controls, and CD1 mice had a strong bilateral response to LPS pre-sensitization to hypoxia–ischemia. Unilateral occlusion by itself is known to produce little to no histological damage (Rice et al., 1981) and reduction on cerebral perfusion (Silverstein et al., 1984). This is likely a result of compensatory mechanisms by the remaining major vessels: contralateral carotid and both vertebral arteries. However, in the presence of hypoxia, this compensatory mechanism is greatly reduced (Silverstein et al., 1984).pH studies following hypoxic–ischemic insult demonstrated a biphasic acidotic shift, where a first pH decrease occurred during the first 30 min following insult and was associated with the ipsilateral area supplied by the MCA, whereas the second shift (60–90 min) also included the adjoining anterior and posterior arteries, these significantly affected the mid-lateral, ipsilateral isocortex, striatum, hippocampus and thalamus (Kendall et al., 2012). With the current 30 min of hypoxia–ischemia, our data concur with the most affected area being supplied by the MCA.

Although some acidosis occurs in the contralateral hemisphere following hypoxia–ischemia, this is primarily present in the areas directly adjacent to the ipsilateral territory, i.e. the anterior (Tuor et al., 1993, Kendall et al., 2012) and posterior cerebral arteries (Kendall et al., 2012). Significantly, this is not observed, at least in short-term period, in the contralateral MCA, arguing against the currently observed pattern of contralateral astrogliosis being a direct result of hypoxia–ischemia. It may be a result of homotypic afferent and efferent projections from roughly the same areas in one cortical hemisphere to another, i.e. damage on ipsilateral side leading to secondary response on the contralateral side, as seen in rats (Moumdjian et al., 1991), and that in mice, the C57BL/6 and CD1 strains are particularly susceptible.

Additionally, we have observed an increased level of GFAP-IR in the FVB naïve strain, which could potentially have an effect in the higher response observed in this particular strain, and therefore future studies should also include assessment of naïve animals as baseline response.

### C57BL/6 as the optimal strain in combined inflammation and hypoxia–ischemia

C57BL/6 and 129SVJ are the most used parent strains in the creation of transgenic mice. Our data show that both strains have minimal response to hypoxia–ischemia. However, C57BL/6 animals have a higher degree of brain injury following LPS pre-treatment compared to 129SVJ animals. This is in concordance with previous findings, where C57BL/6 animals showed a larger vascularised area supplied by the MCA (Maeda et al., 1998). Additionally, 129SVJ mice have reduced ability to recruit immune cells (White et al., 2002, Hoover-Plow et al., 2008) and produce TNFα cytokine (Lambertsen et al., 2002). This makes the use of the 129SVJ strain less than desirable in our model of LPS sensitization to hypoxia–ischemia. BALB/c animals, although responsive to LPS sensitization, only showed a mild non-significant increase in damage. Additionally, there was a baseline response to hypoxia–ischemia, thus making this strain less than ideal for the purpose of LPS-mediated sensitization in order to elicit brain injury. This same principle excludes both CD1 and FVB animals, as brain damage following hypoxia–ischemia was substantial in both strains. C57BL/6 is the most well characterized isogenic strain, allowing better understanding of the response to different stimuli, and demonstrated a robust response to LPS sensitization. This strain would allow a clear comparison between LPS and saline groups, with the added benefit of non-sensitivity to the moderate 30 min 8% oxygen hypoxia–ischemia.

### Conclusion

Both cerebral blood vascularity and immune sensitivity are known factors affecting response to brain injury and appear to be strain-dependent in both the hypoxia–ischemia and in the inflammation-sensitization to hypoxia–ischemia models, with robust FVB and CD1 and moderate BALB/c response in both types of insult. The C57BL/6 and 129SVJ inbred strains appear particularly resistance to hypoxia–ischemia alone. However, LPS proved successful in sensitizing both strains. C5BL/6 and FVB mice showed a saline-mediated increase in inflammatory markers, possibly as a result of stress-associated activation of the immune system, indicating a need for proper consideration of controls when choosing a study design.

## Figures and Tables

**Table 1:** Brain injury score assessment

Score	alphaM immunoreactivity
0	No microglial activation
1	Focal activation
2	Mild diffuse activation, occasional ameboid macrophages present
3	Widespread activation, predominant ameboid macrophages present
4	Tissue loss

Score	Nissl (Cresyl Violet) staining

0	No damage
1	Minimal evidence of damage with no discernible infarct
2	Small infarct (<50%) of the affected brain region
3	Large infarct (>50%) of the affected brain region
4	Total neuronal cell loss

Brain injury score. There is a direct correlation between the level of microglial activation and neuronal cell loss (adapted from (Kendall et al., 2006)).
